# Higher levels of autistic traits associated with lower levels of self-efficacy and wellbeing for performing arts professionals

**DOI:** 10.1371/journal.pone.0246423

**Published:** 2021-02-17

**Authors:** Eleanor Buckley, Elizabeth Pellicano, Anna Remington

**Affiliations:** 1 UCL Centre for Research in Autism and Education (CRAE), University College London, London, United Kingdom; 2 Macquarie School of Education, Macquarie University, Sydney, Australia; University of Warsaw, POLAND

## Abstract

This study sought for the first time to identify the extent to which autistic people are pursuing careers in the performing arts, and to determine the nature of the relationship between individuals’ autistic traits and their reported wellbeing. To address these aims, we recruited a self-selecting, community-based sample of individuals working in the performing arts and invited them to complete an online survey. A total of 1,427 respondents took part. We collected responses on participants’ backgrounds, including diagnostic history as well as measures assessing their level of autistic traits, perceived occupational self-efficacy, quality of life, and mental health. They were also asked open-ended questions about support needed, received, or desired in their workplace. Eleven of the 1,427 professionals (1%) reported a clinical diagnosis of autism. Correlational analyses demonstrated that higher levels of autistic traits were significantly associated with lower levels of quality of life, lower levels of occupational self-efficacy and greater severity of mental health conditions. Almost half the sample of professionals (N = 621; 44%) reported a desire for more employment-based support, and autistic traits were significantly higher in those participants who wanted greater support. Within the community of those working in the performing arts, there are a minority of individuals who are autistic or who have high levels of autistic traits. We have demonstrated for the first time that these individuals may be especially vulnerable to lower wellbeing.

## Introduction

In research, creativity is not something that has been traditionally associated with autism. One key diagnostic criterion for autism–rigid and repetitive behaviours and interests–alongside research showing that autistic people tend to exhibit less flexibility and fluency on creative tasks compared with neurotypical people [1–3], suggest that creativity and out-of-the-box thinking might be challenging for autistic people. This assumption may lead many to think that there are very few autistic people working in the performing arts. Though there has been little systematic investigation into the relationship between autistic traits and creativity [4], recent work showing that autistic people *excel* at producing original output on creative tasks [2, 5, 6] has prompted researchers to rethink these traditional views. Others, too, have recognised that, in practice, there are autistic people with great creative abilities, working across all fields [7–9].

One of many career paths associated with creative talent is that of the performing arts, and “being artistic” has been recognised by worldwide autism experts as a strength of autistic adults [9]. There are many examples of high-profile autistic performing artists, including actors such as Dan Aykroyd, Anthony Hopkins, Paddy Considine and Daryl Hannah, musicians such as Gary Numan, Matt Savage and Derek Paravacini, the opera singer Sophia Grech, and the dancer Philip Martin-Nielson, to name but a few [10–18]. Despite many anecdotal reports, however, no existing research, at least to our knowledge, has examined the experiences of autistic individuals working in the performing arts field. The current study seeks to address this gap directly.

The performing arts is a wide and varied profession but, typically, artists are self-employed and require a broad range of areas beyond their own specialism in order to forge a successful career. Small business and project management skills, including administration, financial management, time management, networking, grant and application writing, arts advocacy and self-promotion, are therefore highlighted as particularly essential to sustaining a career in the performing arts [19]. These skills place considerable demand on people’s executive functions, including flexibility, planning and organization, and on social communication–two areas in which autistic people have particular difficulties [20, 21]. Nevertheless, a recent employment survey for autistic adults revealed that 11% of respondents hoped to work in the arts or acting, demonstrating a clear desire for at least some of the autistic population to pursue careers within this field [22].

The need to support such individuals–and those who are already working in the sector–is borne out by the current employment gap for autistic people. In the UK, only 16% of autistic adults are in full-time paid work and 32% of autistic adults are in any kind of paid work. These estimates are in contrast to 47% of other disability groups, and 80% of the non-disabled population in the UK who are in paid work [22].

For the few autistic adults who have made it into the workplace, there can be many challenges to overcome. Autistic adults can experience difficulties adjusting to new job settings, have communication difficulties with co-workers or supervisors, and may not be offered any workplace adjustments to support them (e.g., mentoring arrangements, sensory adjustments, adjustment to the timetables and tasks) [23–25].

With autistic adults facing potential challenges in the workplace it stands to reason that they may be feeling less confident than their neurotypical peers in their ability to complete tasks related to these environments. In neurotypical adults, self-efficacy–one’s belief in one’s ability to succeed [26]–has found to be positively correlated with self-reported quality of life, and inversely related to severity of mental health traits [27]. Self-efficacy is also positively associated with work-related outcomes such as job performance, job satisfaction, and academic performance [28]. Cognitively-able autistic adults have been shown to have significantly lower self-efficacy in both general and occupational self-efficacy than neurotypical adults [29]. Furthermore, self-efficacy has been shown to be better in workplaces that provide individualised support for autistic employees’ specific needs, but nevertheless remains significantly lower than that of neurotypical individuals [30].

In addition to the challenges often associated with being autistic, many individuals are also neurodivergent in other ways. Neurodivergence is a term used to describe people with atypical neurodevelopment. Around 30% of autistic people have additional intellectual difficulties [31] and many autistic people have at least one co-occurring psychiatric condition [32–35], especially ADHD, which co-occurs in 30%-80% of the autistic population [36, 37]. Individuals with co-occurring autism and ADHD are more likely to have a poorer quality of life than individuals with only one of these conditions [38], and also have lower general self-efficacy [39] and encounter greater difficulties with everyday functioning than autistic people without ADHD [40]. Even further still, approximately 70–80% of autistic children and adults also experience mental health problems [35, 37, 41], with anxiety and depression being the most common [42]. These figures in the autistic population are in stark contrast to estimates of 17% of the adult population in England who meet criteria for a mental health condition at any one time [43]. Alongside poorer mental health, autistic adults also typically report poorer quality of life than that of neurotypical people [44, 45] and autistic traits have been found to be inversely correlated with quality of life [46].

### The current study

Overall, the existing literature paints a picture of a population who may have additional needs but have been thus far overlooked–especially within creative disciplines, such as the performing arts. Yet, the paucity of research in this area means that the extent and nature of these needs, and what career-specific support this population may desire is virtually unknown.

In addition to those who have received, or qualify for, a formal diagnosis, there are also suggestions that the behavioural characteristics extend beyond autism into the general population [43, 47]. Some studies have demonstrated a relationship between behavioural characteristics and objective markers highlighted in studies including diagnosed autistic people. For example, higher (albeit subclinical) autistic traits in individuals (without an autism diagnosis) are related to poorer social cognition and social skills [48] and cognitive and behavioural inflexibility [49]. Furthermore, many autistic adults lack reliable records of developmental history [50], may have been misdiagnosed or missed out on a diagnosis altogether, especially older autistic adults, autistic women and non-binary people [51–54]. For these reasons, the current study therefore examined both the experiences of performing arts professionals who report a formal autism diagnosis and the relationship between level of autistic traits and wellbeing and support needs in the general population of performing arts professionals.

## Method

### Design

The specific aims of this study were threefold. First, we sought to record the experiences of autistic individuals pursuing careers in the arts. Second, we examined the relationship between level of autistic traits and occupational self-efficacy, quality of life, and mental health in performing arts professionals. There is already an established relationship between autism and these variables, and the present study investigated whether this extended to sub-clinical levels of autistic traits. Third, we investigated the relationship between individuals’ level of autistic traits and their support needs.

To address these aims, an online questionnaire was completed by adults who worked in the performing arts. We predicted that individuals with higher levels of autistic traits would have lower self-efficacy, poorer mental health, lower quality of life, and would be more likely to have needed and to desire occupational support than those with lower levels of autistic traits.

### Participants

A large number (N = 1,427) of performing arts professionals based in the United Kingdom (UK) completed an online questionnaire powered by Qualtrics [55]. Demographic information is shown in Table 1. Of the performing arts professionals, there was a nearly even gender split with slightly more female participants (55%) and the majority of respondents reported to be of White ethnic background (89%). Professionals reported having worked in the performing arts for an average of 10 years (ranging from under 1 year to over 20 years; see Table 1). Participants were recruited through convenience sampling methods, whereby the online questionnaire was advertised through targeted emails to performing arts groups including members of the UK performing arts union, Equity, promotion on social media, and word of mouth.

**Table 1:** Performing arts professionals’ characteristics.

Characteristic	Performing arts professionals N = 1,427
**Age**	
Mean, years (SD)	43.9 (15.0)
Median, years	42
Range	18–89
18–19, years	1 (<1%)
20–29, years	293 (21%)
30–39, years	349 (24%)
40–49, years	262 (18%)
50–59, years	258 (18%)
60–69, years	188 (13%)
70–79, years	63 (4%)
80–89, years	9 (<1%)
Prefer not to say	4 (<1%)
**Gender**	
Female	784 (55%)
Male	622 (44%)
Non-binary or other	12 (1%)
Prefer not to say	9 (1%)
**Ethnicity**	
White	1,270 (89%)
Black	41 (3%)
Asian	16 (1%)
Any other ethnic group	83 (6%)
Prefer not to say	17 (1%)
**Highest level of education completed or in progress**^a^	
No schooling completed	8 (1%)
GCSEs	46 (3%)
BTECs	22 (2%)
A Levels or IB	71 (5%)
Trade, technical, or vocational training	152 (11%)
Undergraduate degree	644 (45%)
Postgraduate degree	342 (24%)
Other	136 (10%)
Prefer not to say	6 (<1%)
**Length of time working in the performing arts**	
Under 1 year	55 (4%)
1–5 years	229 (16%)
6–9 years	166 (12%)
Over 10 years	973 (68%)
Prefer not to say	4 (<1%)

^a^Notes. GCSE stands for General Certificate of Secondary Education, BTEC stands for Business and Technology Education Council diploma, A Level stands for Advanced Level, IB stands for International Baccalaureate.

### Measures

The online questionnaire contained six sections, which took approximately 25–30 minutes to complete.

1. Part 1 of the questionnaire began with a series of demographic items, including participant age, gender, ethnicity, and highest level of education. Participants were then asked to identify whether they were currently working in the performing arts.
2. Part 2 of the questionnaire contained a bespoke scale to measure occupational self-efficacy for performing arts professionals. The bespoke scale was designed to address the unique demands of performing arts careers [19]. It was based on Bandura [56] but was adapted specifically to target professionals’ perceived confidence when performing activities associated with their performing arts careers. An initial focus group was held with 6 performing arts professionals to help identify appropriate items to be included on the scale. Following Bandura [56], the self-efficacy scale contained 24 items where participants could respond to each item with a score ranging from 0 (“not at all confident”) to 10 (“extremely confident”). Items used in the scales included, among others, “Interview / audition for roles”, “Fully understand all instructions given to me”, and “Get a colleague or peer to help me if I have difficulty interacting with others at my workplace”. Participants could select ‘not applicable’ to individual items on the self-efficacy scale which were not relevant to their careers. Scores from all items completed were averaged to yield a mean self-efficacy score. Higher scores reflected greater occupational self-efficacy. The scale showed excellent internal consistency (Cronbach’s alpha = .94) [see S1 File for the full scale, and analysis concerning the reliability of the scale for participants with missing values].
3. Part 3 of the questionnaire contained three closed questions about support in relation to professionals’ workplaces: Question 1: “Have you ever needed extra support for your performing arts career but did not receive it?”; Question 2: “Have you ever received extra support for your performing arts career?”; Question 3: “Would you like extra support for your performing arts career?”. Participants could answer “yes”, “no”, or “I do not wish to answer this question” to each question. Participants were then asked to provide details about the support needed, received or desired in an open comment box.
4. Part 4 of the questionnaire asked participants to provide details regarding whether they had received clinical diagnoses of autism, mental health conditions/neurological conditions, and/or a specific learning difficulty (e.g., dyslexia). If participants reported that they had any condition that was contained within those categories, they were then asked, “Do you feel that your condition(s) impacts on different aspects of your career? If yes, please go into detail here” and an open comment box was available for participants to provide details.
5. Part 5 of the questionnaire contained several established psychological measures to examine traits related to various neurological conditions and current levels of wellbeing.

The *Subthreshold Autism Trait Questionnaire* (SATQ; [43]) was created to assess a broad range of subthreshold traits of autism in a general population. Unlike the Autism Quotient (AQ; [57]) which was designed to highlight presentation of symptoms that are characteristic of Asperger Syndrome, the SATQ provides a measure of a broader range of autistic traits [58, 59]. The SATQ has 24 items, and has good internal consistency and reliability (Cronbach’s alpha coefficient = .73, test-retest reliability = .79; [43]); in the present study, Cronbach’s alpha coefficient = .83). The SATQ asks participants to respond to statements such as “I sometimes take things too literally, such as missing the point of a joke or having trouble understanding sarcasm” with a 4-point scale ranging from “false, not at all true” (score of 0) to “very true” (score of 4). High scores on the SATQ reflect high levels of autistic traits. There is currently no cut-off point for this scale associated with receiving an autism diagnosis.

The *Patient Health Questionnaire depression scale* (PHQ-8; [60]) is an 8-item questionnaire, which assesses traits of depression. It asks participants to rate how often in the past two weeks they have had particular feelings or acted in a certain way, for example, “feeling down, depressed, or hopeless” and “trouble concentrating on things, such as reading the newspaper or watching television” on a 4-point scale ranging from “not at all” (score of 0) to “nearly every day” (score of 3). Higher scores reflect greater severity of depression. The PHQ-8 cut-off point (scores of 10 or greater) has a sensitivity of 88% and a specificity of 88% for detecting major depression [60], good internal consistency (Cronbach’s alpha coefficient = .89; [61]) and, regardless of diagnostic status, scores above cut-off point typically represent clinically-significant depression [62].

The *Generalised Anxiety Disorder scale* (GAD-7; [63]) assesses traits of anxiety and asks participants how often over the past two weeks have they been bothered by feelings such as “feeling nervous, anxious, or on edge” and “becoming easily annoyed or irritable”. Participants respond to seven questionnaire items with a 4-point scale ranging from “not at all” (score of 0) to “nearly every day” (score of 3), higher scores reflect greater severity of anxiety. The cut-off point for the GAD-7 (scores of 10 or greater) has a sensitivity of 89% and specificity of 82% for detecting generalized anxiety disorder and good internal consistency (Cronbach’s alpha coefficient = .89; [63]).

The *World Health Organization Adult ADHD Self-Report Scale* (ASRS; [64]) was used to assess traits of ADHD. Participants were asked to respond to 6 questionnaire items (e.g., “How often do you have difficulty getting things in order when you have to do a task that requires organization?”) using a Likert scale ranging from “never” (score of 0) to “very often” (score of 4). Higher scores reflect higher ADHD symptom severity. The ASRS screener [64] was scored in line with the recommendations of [65] to be consistent with criteria for ADHD described in the DSM-5. The screener with updated scoring has a sensitivity of 80% and specificity of 90% for identifying people who have a diagnosis of ADHD [65] and good internal consistency (Cronbach’s alpha coefficient = .95; [66]).

The World Health Organization abbreviated version of the WHOQOL-100 quality of life assessment [67]. The WHOQOL-BREF contains 26 items (e.g., “how satisfied are you with your ability to perform your daily living activities?”), which measure four domains of quality of life (physical, psychological, social, environment). The WHOQOL-BREF contains several sections that are all scored differently. Overall, higher scores on the four domains of the WHOQOL-BREF reflect greater quality of life within those specific domains. The WHOQOL-BREF has been shown to be comparable to the WHOQOL-100 in having excellent ability in discriminating between ill and well respondents, good internal consistency (Cronbach’s alpha coefficients for the four domains: Physical = .87; Psychological = .74; Social = .55; Environment = .74) and high test-retest reliability across all four domains [68].

6 Finally, in Part 6, participants could opt to make any extra remarks about their career or thoughts about the questionnaire in one final open comment box.

### Procedure

Ethical approval was obtained from UCL Research Ethics Committee. The online questionnaire was anonymous. Given that we were particularly interested in relationships between measures, only respondents who completed all six parts of the questionnaire were included in analysis.

### Data analysis

#### Quantitative analyses

First, correlational analyses were performed to investigate the extent and nature of any relationships between levels of autistic traits (indexed by SATQ scores) and variables such as self-efficacy, quality of life, and severity of mental health conditions. Not all variables were normally distributed; we therefore used Spearman’s rank correlation coefficients for such variables.

Next, to investigate the relationship between individuals’ autistic traits and their perceived need for support, the professionals were divided into quartiles based on their SATQ scores. The data from the upper and lower quartiles were then compared in an extreme-groups analysis (EGA) to enable us to examine whether those with higher levels of autistic traits were more likely to have needed, received, or desired support than those with lower levels of autistic traits. All analyses were performed using IBM SPSS version 22 software [69].

A *p*-value of 0.05 was set, and due to the high number of comparisons the Holm-Bonferroni method was used to calculate adjusted alpha levels for each set of analyses to control the family-wise error rate [70].

#### Qualitative analyses

To understand the views and experiences of performing arts professionals with regard to the support that they have received and their perceived support needs, participants’ open-ended responses were analysed using thematic analysis, as detailed by [71]. The transcripts were analysed from an inductive (bottom-up) perspective where themes were created within a ‘contextualist’ method of critical realism [72]. The first and last authors carried out the thematic analysis and approached the analysis from the perspective of psychology researchers who have not worked in the performing arts and do not identify as autistic, and so analysed the data from the perspective of outside interpreters. Analyses were performed using NVivo version 11 software.

## Results

### Quantitative analysis

#### Neurodivergence

The first aim of this study was to understand the extent to which autistic individuals are pursuing careers in the performing arts. There were eleven professionals (1%) who reported a clinical diagnosis of autism. The overall average score on the Subthreshold Autistic Traits Questionnaire (SATQ) for professionals was 16.84 (*SD* = 8.86). These scores are lower than the population average of 23, taken from an American student population of 1,709 [57]. Nevertheless, 50 professionals (4%) scored more than 2 SD above the mean score on the SATQ, suggestive of elevated levels of autistic traits. Overall, few participants reported a clinical diagnosis of ADHD (1%, n = 9), and 3% (n = 41) of performing arts professionals scored above the ASRS threshold for ADHD [65]. The percentages from both the screener and the reported clinical diagnoses are consistent with prevalence estimates of between 1–7% of the European population having ADHD [73]. We found that eight percent (n = 112) of professionals reported a specific learning disability, of which 79 (71%) reported a diagnosis of dyslexia.

#### Quality of life

Quality of life scores for the professionals were within population norms [68]. WHOQOL-BREF domain scores for all participants ranged between 4 and 20: Physical: M = 15.9 (SD = 2.5), Psychological: M = 14.0 (SD = 3.0), Social: M = 13.4 (SD = 4.1), and Environment: M = 15.3 (SD = 2.9).

#### Mental health

We found that fifteen percent (n = 214) of professionals reported a clinical diagnosis of depression, but almost one third of the group (n = 434; 30%) of professionals scored above the PHQ-8 cut-off point, indicating clinically significant levels of depression. These percentages are much higher than the US population-based study of over 198,000 participants, which recorded prevalence of 8.6% scoring over the cut-off point for clinical levels of depression [74].

A similar picture was evident with respect to anxiety: 13% (n = 190) of performing arts professionals reported a clinical diagnosis of anxiety, but 26% (n = 372) scored above the GAD-7 cut-off point, indicative of clinically significant levels of anxiety. Again, these percentages are much higher than population norms: 5% scored over the cut-off point in a German population-based study of over 5,000 participants [75].

#### Self-efficacy

Professionals experienced high self-efficacy with respect to their profession (see Table 2 for mean scores). Professionals were most confident at taking part in performances (M = 9.00, SD = 1.50) and least confident at networking to secure future opportunities (M = 5.60, SD = 2.70).

**Table 2:** Mean scores and standard deviations for items on the performing arts professionals’ occupational self-efficacy scale.

Self-efficacy item	M (SD)	Range
1. Fully understand what I am required to do to be proactive in my career	7.5 (2.1)	0–10
2. Motivate myself to work (e.g. apply for roles, rehearse)	7.5 (2.1)	0–10
3. Fully understand all instructions given to me	8.3 (1.8)	0–10
4. Structure my time to manage my workload	7.3 (2.2)	0–10
5. Keep to external deadlines	8.6 (1.6)	0–10
6. Concentrate when at work	8.6 (1.6)	0–10
7. Remember information presented at work or in books	8.0 (1.8)	0–10
8. Take good notes during instruction from others	8.0 (1.9)	0–10
9. Independently study or research	8.1 (1.9)	0–10
10. Complete classes or workshops that I have signed up for	8.6 (1.8)	0–10
11. Participate in group exercises	8.0 (2.1)	0–10
12. Work with others to achieve a joint goal	8.8 (1.5)	1–10
13. Share my ideas in group discussions	8.2 (1.9)	0–10
14. Lead or coordinate my peers / colleagues in group work	7.5 (2.2)	0–10
15. Interview / audition for roles	7.5 (2.3)	0–10
16. Prepare for performances (this includes technical work, rehearsals, etc. as applicable)	8.8 (1.5)	1–10
17. Take part in performances	9.0 (1.5)	0–10
18. Make phone calls to people I don’t know (for work-based purposes, e.g. to hire equipment)	6.8 (2.8)	0–10
19. Socialize with others in my workplace	7.5 (2.3)	0–10
20. Ask for help with my work (if required) from a colleague or peer	7.4 (2.3)	0–10
21. Ask for help with my work (if required) from an employer or member of production team	7.6 (2.2)	0–10
22. Get a colleague or peer to help me if I have difficulty interacting with others at my workplace	6.0 (2.8)	0–10
23. Get an employer or member of my production team to help me if I have difficulty interacting with others at my workplace	5.7 (2.9)	0–10
24. Network to secure future opportunities	5.6 (2.7)	0–10
**Total**	**7.8 (1.3)**	**3–10**

#### Support

Almost one quarter (24%, n = 348) of professionals reported that they had received occupational support. More than one third (37%, n = 529) of professionals reported that they had needed but not received support in their careers, and just under one half of the sample (44%, n = 621) reported that they would like to receive support in the future.

#### The relationship between autistic traits and other variables

As shown in Table 3, there were significant correlations between all variables, all of which survived corrections for multiple comparisons using the Holm-Bonferroni method [70]. As expected, professionals with elevated levels of autistic traits (i.e., high SATQ scores) had lower perceived self-efficacy as well as lower quality of life (as indexed by scores on all four domains of the WHOQOL-BREF). We also found significant correlations between SATQ scores and scores on measures of co-occurring conditions. Higher levels of autistic traits were associated with elevated levels of anxiety (GAD-7 scores), depression (PHQ-8 scores) and ADHD symptomology (ASRS scores). We also show in Table 3 partial correlations between autistic traits, occupational self-efficacy, the four domains of the WHOQOL-BREF, PHQ-8, GAD-7, and ASRS scores adjusted for gender and age as covariates. We found significant correlations between all of the variables.

**Table 3:** Correlation matrices for performing arts professionals’ scores on occupational self-efficacy, SATQ, WHOQOL-BREF domains, PHQ-8, GAD-7, ASRS, age, and gender.

		SATQ	Self-efficacy	WHOQOL physical domain	WHOQOL psychological domain	WHOQOL social domain	WHOQOL environment domain	PHQ-8	GAD-7	ASRS	Age
SATQ	r_s_	1.00	-.395**	-.327**	-.394**	-.281**	-.288**	.387**	.326**	.295**	
	Sig.		< .001	< .001	< .001	< .001	< .001	< .001	< .001	< .001	
Self-efficacy	r_s_	-.414**	1.00	.340**	.437**	.326**	.351**	-.316**	-.264**	-.278**	
	Sig.	< .001		< .001	< .001	< .001	< .001	< .001	< .001	< .001	
WHOQOL physical domain	r_s_	-.324**	.333**	1.00	.593**	.434**	.584**	-.584**	-.471**	-.362**	
	Sig.	< .001	< .001		< .001	< .001	< .001	< .001	< .001	< .001	
WHOQOL psychological domain	r_s_	-.404**	.458**	.590**	1.00	.576**	.611**	-.700**	-.626**	-.434	
	Sig.	< .001	< .001	< .001		< .001	< .001	< .001	< .001	< .001	
WHOQOL social domain	r_s_	-.298**	.329**	.434**	.562**	1.00	.508**	-.439**	-.390**	-.272**	
	Sig.	< .001	< .001	< .001	< .001		< .001	< .001	< .001	< .001	
WHOQOL environment domain	r_s_	-.340**	.399**	.584**	.619**	.503**	1.00	-.501**	-.473**	-.383	
	Sig.	< .001	< .001	< .001	< .001	< .001		< .001	< .001	< .001	
PHQ-8	r_s_	.387**	-.361**	-.586**	-.697**	-.437**	-.505**	1.00	.784**	.496**	
	Sig.	< .001	< .001	< .001	< .001	< .001	< .001		< .001	< .001	
GAD-7	r_s_	.344**	-.324**	-.494**	-.640**	-.379**	-.488**	.783**	1.00	.485**	
	Sig.	< .001	< .001	< .001	< .001	< .001	< .001	< .001		< .001	
ASRS	r_s_	.320**	-.319**	-.348**	-.416**	-.257**	-.386**	.484**	.482**	1.00	
	Sig.	< .001	< .001	< .001	< .001	< .001	< .001	< .001	< .001		
Age	r_s_	-.141**	.237**	.03	.209**	.056*	.281**	-.281**	-.294**	-.291**	1.00
	Sig.	< .001	< .001	.19	< .001	.04	< .001	< .001	< .001	< .001	
Gender	r_s_	.175**	.01	.02	.05	-.04	.04	-.072**	-.114**	-.055*	.161**
	Sig.	< .001	.73	.41	.05	.14	.14	.01	< .001	.04	< .001

Correlations presented in the lower diagonal are the raw correlations between variables, correlations presented in the upper diagonal of the table are partial correlations between variables adjusted for chronological age and gender. Numbers with two asterisks ** beside them indicate a significant result.

Notes. Measures included in the table are: The Subthreshold Autism Trait Questionnaire (SATQ; [43]) measuring autistic traits. The World Health Organization abbreviated version of the WHOQOL-100 quality of life assessment (The WHOQOL-BREF; [64]) measuring the 4 domains of quality of life (physical, psychological, social, and environment). The Patient Health Questionnaire depression scale (PHQ-8; [60]) measuring depression traits. The Generalised Anxiety Disorder scale (GAD-7; [63]) measuring anxiety traits. The World Health Organization Adult ADHD Self-Report Scale (ASRS; [64]) measuring ADHD traits.

#### The relationship between high and low autistic traits and support

We hypothesized that those with higher levels of autistic traits were more likely to have needed and desired support than those with lower levels of autistic traits. Percentages in parentheses indicate the proportion of participants who responded “yes” to each question item.

We began by comparing the frequency of individuals reporting having previously received support in the high and low autistic traits groups (see analysis section of method for details of group creation). As shown in Table 4, professionals in the high autistic trait group were just as likely to have received support (24%) as those in the low autistic traits group (22%). Members of the high autistic traits group were significantly more likely, however, to report having needed support but not having received it (40%) than members of the low autistic traits group (34%). Analyses also revealed a significant group difference in terms of how many of them desired support in the future: professionals with high autistic traits were more likely to desire support in the future (49%) than those with low autistic traits (38%).

**Table 4:** Extreme groups analyses using upper and lower quartiles of the performing arts professionals’ SATQ scores to compare ‘high autistic traits’ and ‘low autistic traits’ groups.

	Pearson Chi Square Value	Degrees of freedom	Significance	Effect size (Cramer’s V)
Received support	0.79	1	.374	.034
Needed support	4.73	1	.030	.084
Desire future support	11.48	1	.001	.131
Scoring ≥ clinical significance on PHQ-8	130.29	1	<.001	.427
Scoring ≥ clinical significance on GAD-7	72.44	1	<.001	.319
Scoring ≥ clinical significance on ASRS	9.19	1	.002	.113

Next, we examined the frequency of individuals in the high and low autistic traits groups scoring at clinically significant levels for depression, anxiety and ADHD traits. Professionals in the high autistic traits group were significantly more likely to meet clinically significant thresholds on all of the measures (PHQ-8, GAD-7, ASRS) in comparison to the low autistic traits group (odds ratio for high autistic traits group scoring at clinical significance on PHQ-8 = 8.16; odds ratio for high autistic traits group scoring at clinical significance on GAD-7 = 4.73; odds ratio for high autistic traits group scoring at clinical significance on ASRS = 4.69).

### Qualitative results

**Fig 1:**
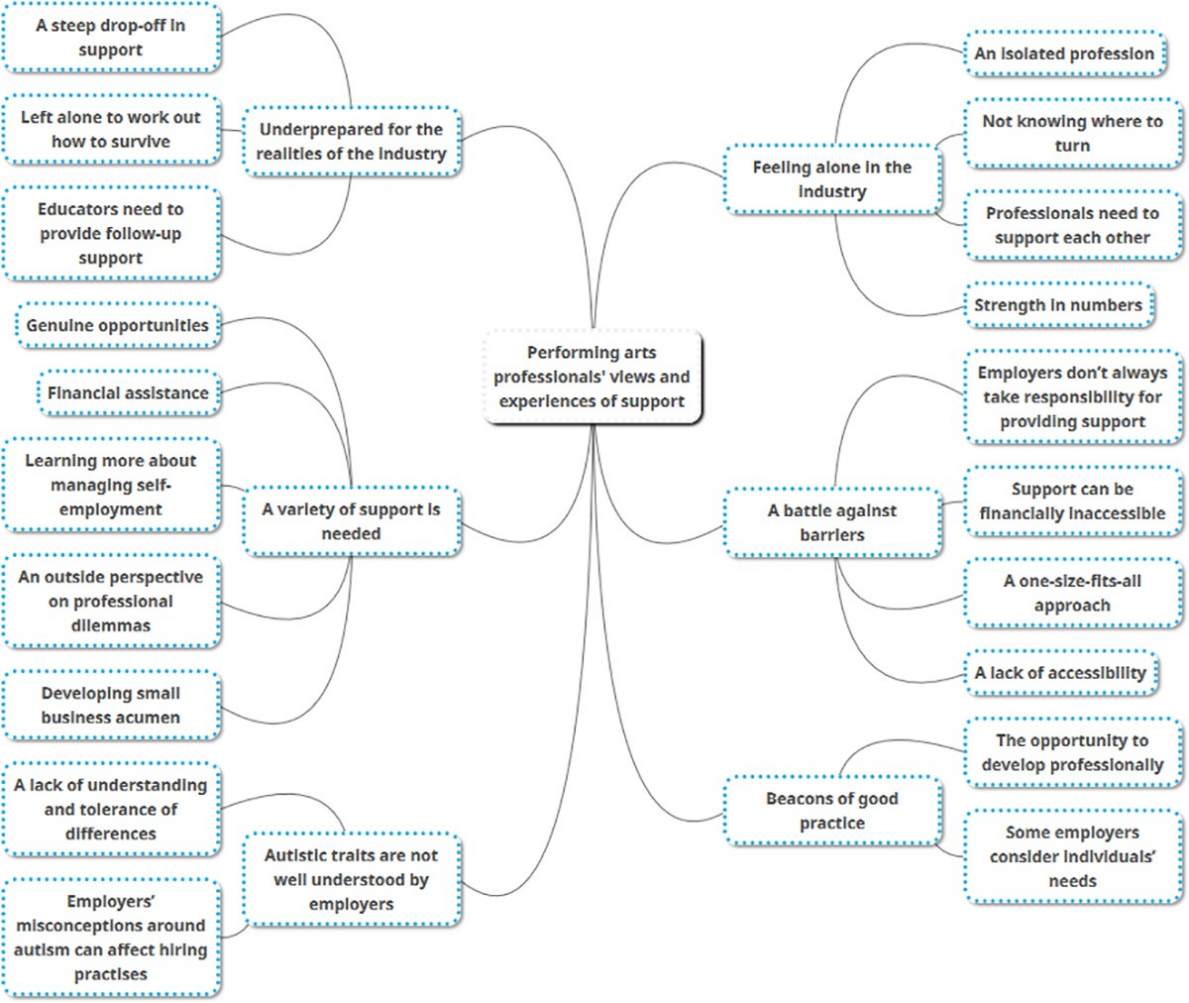
The views of performing arts professionals on support received/desired and their self-identified support needs: Themes and subthemes.

In total, 759 professionals (53%) responded to the open question asking about whether they had previously needed, asked for, or would like support in their workplace setting. The aim of the qualitative analysis was to explore experiences of support across all performing arts professionals who completed the questionnaire. Not all support needs will be related to autistic traits or specific to autistic people, so we felt it was important to include a broad spectrum of experiences of support. Alongside analysing all of the survey participants’ comments together, we also sought to identify themes that were unique to participants who were autistic. Examination of their views and experiences showed that they were consistent with the remaining questionnaire respondents, so, for the sake of brevity, the themes are therefore presented together. We identified six main themes from all participants, with one theme unique to the autistic group. These are presented below along with their subthemes, which are italicised in the text (see Fig 1 for all themes and subthemes). All quotations are labelled with their participant number (abbreviated to ‘ppt’).

#### Feeling alone in the industry

A recurrent sentiment expressed by participants was that there was no support available to them and that they were in *an isolated profession*: “The industry doesn’t really offer support. You are on your own” [ppt 493]. Some participants also highlighted that they *didn’t know where to turn* for support, if it did indeed exist: “Don’t know who to ask or what support is available really” [ppt 725]. Many professionals felt that they were disconnected from their peers in the profession and spoke of a desire for *professionals to support each other* through forming mutual support groups: “There should be more open networking events and support groups for people in the industry” [ppt 1196]. There was a sense that if support groups could be set up and professionals had spaces where they could come together outside of the workplace the *strength in numbers* would be of benefit to many: “Keeping a community together to be secure in the industry” [ppt 93].

#### Underprepared for the realities of the industry

Professionals frequently commented on how ill-equipped they felt for being alone in the working world after finishing training at a performing arts school. They spoke of the *steep drop off in support* from what they had received during their higher education to what they received in their professional lives: “It was shocking leaving drama school and going into the industry. It feels like all the support disappears once you graduate” [ppt 1224]. Many felt that had been *left on their own to work out how to survive* in the industry with no one to turn to for advice or support when challenges arose: “As soon as I left training I felt very alone in the industry and had to figure it all out as I go” [ppt 1395]. Professionals urged performing arts schools to *provide follow-up support* for recent graduates, to help ease them into careers in their chosen profession, and check on their progress and whether they needed support: “More personal support after graduating. Maybe a one-to-one to see how we progress with our careers after training” [ppt 1394].

#### A battle against barriers

Professionals described many barriers to accessing support in their workplaces. They spoke of feeling as if they just had to “get on with it” and that often support was not provided because *employers don’t always take responsibility for providing support* and that they should be able to cope with whatever they were dealing with. If not, then the fault and the ability to solve the problem was perceived to lie with the employee rather than the employer: “There seems to be an expectation of being very well, very positive and able to deal with anything thrown at you” [ppt 1183]. Financial constraints were highlighted as another barrier to support. Professionals stated that their workplaces were not always in a financial position to provide sufficient support. Moreover, professionals who were self-employed or between jobs also spoke of the financial hardship for many, and that accessing support was simply *financially out-of-reach*: “The only way to receive extra support is to pay for it and this is not always realistic when you are a self-employed/out of work actor” [ppt 1324]. Professionals touched on the idea of how their workplaces employed *a one-size-fits-all approach* to aspects of the profession, and they spoke of feeling penalized for having cognitive differences that meant they may need extra time to prepare for auditions, which employers were not recognizing: “I know a lot of actors are dyslexic and it’s so difficult to learn lines for auditions in 1 or 2 days or sometimes just with an evening’s notice, especially when working day jobs. I know that’s the way the industry is, but I feel at a real disadvantage” [ppt 1349]. This sentiment around *a lack of accessibility* was echoed by many other participants who raised this concern: “My greatest issue with the industry though is the manner in which a lot of auditions are set up. You get no warning or notice” [ppt 589].

#### A variety of support is needed

Professionals described the spurious nature of some purported support, such as workshops. Workshops can be opportunities to hone skills specific to the profession and are also a source of potential networking and even future job opportunities. A recurring sentiment about these workshops from participants was that often workshops did not truly offer *genuine opportunities* to learn new skills, but were rather held for the financial benefit of those hosting the workshop: “Increasingly, workshops with casting directors are just actors paying so that they can be seen, as opposed to learning new skills” [ppt 1324]. Professionals spoke of the financial hardship that can accompany a career in the performing arts, and so for many receiving *financial assistance* was a priority. This assistance would be to cover living expenses when unemployed (“Some kind of income protection insurance would have been helpful–all time taken off was unpaid, due to self-employment” [ppt 1183]), and also to help support work outside of traditional employment (‘‘funding towards creative projects” [ppt 1292]). Professionals wanted sources of general advice, so that they could *learn more about managing their self-employment* and knowing what they were entitled to: “It would be great if there was some kind of helpline I could call to see what my rights and benefits are” [ppt 1179]. Many also hoped to find a source of individualized support and guidance, to help them further their careers and gain *an outside perspective on professional dilemmas*: “Free honest guidance about what’s best for the next step in my career” [ppt 621]. More specifically, professionals spoke of wanting mentorship but were unsure of how to find this: “I would really like a mentor, and don’t know how to get one” [ppt 750]. Another area where support was desired was in helping professionals *developing small business acumen*. They spoke of needing skills outside their area of expertise in order to manage their self-employment: “Creating and managing a website, keeping on top of accounts, self-promotion–these are things that do not come naturally” [ppt 357].

#### Beacons of good practice

Despite raising issues around the low quality and quantity of support available to professionals, some participants spoke of feeling well supported when they had needed extra help. Some professionals felt that they had been given *the opportunity to develop professionally*, both through learning occupation-specific skills and receiving guidance and advice from others in the profession: “Supported my [theatre director] training and mentored me” [ppt 474]. Others spoke of being in a supportive workplace environment, where their *individual needs were considered by their employers* and they were able to seek extra support for physical or mental health concerns. Examples of support received were being able to take time out from work when needed and being offered in-house counselling: “I [did] receive great support from the opera company when I lost my voice. They let me take time out and then come back” [ppt 708].

#### Autistic traits are not well understood by employers

Autistic professionals described encountering *a lack of understanding and tolerance of differences* from others: “People sense I’m different and don’t want to invest in me as readily” [ppt 1152]. Many were concerned about *employers’ misconceptions around autism* and how this may affect their job applications and time in the workplace: “I feel Asperger’s Syndrome is still not properly understood, at least at higher levels in the business and this can adversely affect people’s perception of me when applying for work” [ppt 497].

## Discussion

The results of this study have demonstrated for the first time that those who have higher levels of autistic traits and are pursuing careers in the performing arts may be especially vulnerable to lower occupational self-efficacy and higher rates of mental health issues than those with lower levels of autistic traits. These individuals are also more likely to desire employment-based support than individuals with lower levels of autistic traits, indicating that this may be a population who needs sustained support.

The overall mental health of performing arts professionals in this sample is far lower than expected for the general population. In England, 17% of the adult population meet criteria for a mental health condition at any one time [43], with the majority suffering from depression or anxiety. *Twice* as many professionals scored above threshold for clinically significant levels of depression and anxiety on the screening tools as those reporting clinical diagnoses of these conditions. There is limited published research on the quality of life and mental health of the performing arts population, although it is anecdotally acknowledged that there is a high incidence of poor mental health in the arts [76, 77].

One potential explanation for the high rates of mental health issues in this group is the uncertainty surrounding employment. Long-term employment in the arts is rare, many employers engage in a project-based system of hiring, which leaves professionals constantly seeking new employment and often having to manage periods of unemployment [78]. It is well-documented that unemployment is linked to poor mental health [79] and low quality of life [80], and therefore job instability in this population may be an important contributing factor to these results. Another, related explanation is the perceived lack of occupational support within careers in the performing arts. Many of the professionals’ comments centred on feeling unsupported and isolated in their careers. These experiences of loneliness in their professional lives could well relate to their reported low quality of life and poor mental health [81].

A lack of job stability and the constant pressure to gain new employment are likely to be especially challenging for those with a diagnosis of autism or elevated autism traits. This might be particularly hard for autistic people to manage, due to difficulties dealing with uncertainty and communication with neurotypical others. There may be high anxiety around auditions, and they may be struggling with aspects of job interviews such as small talk. As expected, and consistent with existing work [44, 45], performing arts professionals with higher levels of autistic traits were more likely to report poor quality of life across all domains than professionals with lower levels of autistic traits. We also found that individuals with higher levels of autistic traits were significantly more likely also to have poorer mental health and ADHD symptomatology than those with lower levels of autistic traits. This is concerning, but perhaps unsurprising. Research suggests that a higher percentage of the autistic population have mental health and co-occurring psychiatric conditions than the general population [41, 43, 82].

The results indicating that quality of life and mental health are poorer for those with higher levels of autistic traits than for those with lower levels of autistic traits could be due to many factors. One such factor may be participants’ experiences of their workplace environments. It is well known that in the workplace, autistic people often receive insufficient reasonable adjustments, poor mental health support, and report anxiety in relation to their working environment [23–25, 83]. The comments made by formally diagnosed autistic professionals in this study described employers not being understanding or accommodating of autistic characteristics, with this lack of accommodation for behavioural differences perceived to be contributing to their lower quality of life and mental health. It may be important to encourage employers to learn more about autism and autistic traits, so that workplaces can be more accepting and accommodating of difference.

Consistent with our hypotheses, we found that performing arts professionals with higher levels of autistic traits have lower occupational self-efficacy, than those with lower levels of autistic traits. These findings reflect research that has found autistic people to have lower occupational and general self-efficacy than non-autistic people [29] and indicates that those with subclinical levels of autistic traits may also be experiencing challenges [47, 84]. Lower self-efficacy may be influenced by the reported lack of understanding from employers, who may not trust that disabled or neurodivergent employees can perform the job equally as well, which could mean less frequent opportunities to advance and build skills [85–87]. We note that age was significantly positively associated with occupational self-efficacy and significantly inversely correlated with autistic traits, which may be explained by professionals developing more career-related skills and coping mechanisms as they age [88, 89].

Performing arts professionals with higher levels of autistic traits were more likely than those with low levels of autistic traits to report that they have previously needed employment-based support and not received it, and they were also more likely to desire support in the future for their career. The types of support that these professionals want are similar to those desired by all of the professionals surveyed: help with developing small business acumen, financial assistance, networks to connect professionals together, sources of general advice, alongside more tailored advice, such as mentoring. These skills and support needs are recognised aspects of performing arts careers [19], and future research should examine types of support that may be particularly effective for those with elevated levels of autistic traits. Given that there were also significant associations between autistic traits, mental health symptomology and ADHD traits, future work must take into consideration what other factors may be driving this need for support.

One strength of this study is that it is the first to use a large UK-based sample of performing arts professionals to examine individuals’ occupational confidence and their perspectives on support available in the performing arts. It is also the first time that the relationship between autistic traits and these factors has been examined in this group. There are 296, 000 people who are estimated to work in the performing arts in the UK [90], so respondents comprise less than 1% of that figure. However, the study was advertised widely through social media and emails to a diverse network of performing arts groups and individuals and the proportions of many of the demographic variables measured are similar to UK population estimates. The distribution of genders and ethnicities of the participants in this study are reflective of distributions recorded by UK census figures [91]. It is estimated that autistic people form 1.1% of the UK population [50, 92] in this study autistic people formed 1% of the professional sample. The figures of autistic professionals within this sample therefore reflect UK prevalence estimates.

This study, however, it not without its limitations. First, the method of extreme groups analyses, which was used to examine the difference in levels of need for support between those with higher and lower autistic traits, has been criticised for potentially falsely inflating the power of an analysis and therefore increasing the chances of Type II error [93]. However, we are confident that this is not the case of our study since the samples used for analysis (n = 714) were large, thus reducing the chance of making said Type II error. Second, although the survey methods used in this study meant that the study was accessible and we were able to recruit a large number of participants, all of the measures were self-report and relied on participants to be honest.

In conclusion, the performing arts is a profession that requires workers to develop broad skills in business acumen, frequently manage self-employment, and consistently seek new work in a project-based hiring system. The unique demands of this industry mean that the majority of workers are under constant pressure and leave many desiring career-based support. These findings provide an initial investigation into autistic traits and their relationship with occupational self-efficacy, mental health, quality of life, and support needs for performing arts professionals. The results highlight that those with higher levels of autistic traits working in the performing arts may be particularly vulnerable to low occupational self-efficacy and wellbeing and are more likely to have needed and desire employment-based support. This research has contributed to understanding the experiences of performing arts professionals in the UK, and revealed the increased need for support in workers with elevated levels of autistic traits. Future research should further examine the specific support needs of this group, alongside investigating whether those with autism diagnoses are having similar experiences.

## Supporting information

S1 File Containing the full ‘performing arts occupational self-efficacy scale’; testing the reliability of the ‘performing arts occupational self-efficacy scale’; and examining the influence of autistic participants on results. (PDF)
