# Supplementary material for: Higher levels of autistic traits associated with lower levels of self-efficacy and wellbeing for performing arts professionals
Source: PLoS One. 2021 Feb 17;16(2):e0246423. doi: 10.1371/journal.pone.0246423 (PMC7888677; doi:10.1371/journal.pone.0246423)
Supplement: S1 File — (PDF) [file pone.0246423.s001.pdf]

S1 File

1. The ‘Performing Arts Occupational Self-Efficacy Scale’

This portion of the questionnaire is designed to help us understand challenges for those working in the performing arts.

Please rate how confident you are performing the activities listed below by selecting the appropriate number from 0 to 10:

0 = Not at all confident

5 = Moderately confident

10 = Extremely confident

[Participants are presented with a number scale that allows them to select a whole number between 0 and 10 for each item, they can also opt out of answering individual items on the scale that are not relevant to their career by selecting “not applicable” instead]

1. Fully understand what I am required to do to be proactive in my career

2. Motivate myself to work (e.g. apply for roles, rehearse)

3. Fully understand all instructions given to me

4. Structure my time to manage my workload

5. Keep to external deadlines

6. Concentrate when at work

7. Remember information presented at work or in books

8. Take good notes during instruction from others

9. Independently study or research

10. Complete classes or workshops that I have signed up for

11. Participate in group exercises

12. Work with others to achieve a joint goal

13. Share my ideas in group discussions

14. Lead or coordinate my peers / colleagues in group work

15. Interview / audition for roles

16. Prepare for performances (this includes technical work, rehearsals, etc. as applicable)

17. Take part in performances

18. Make phone calls to people I don't know (for work-based purposes, e.g. to hire equipment)

19. Socialize with others in my workplace

20. Ask for help with my work (if required) from a colleague or peer

21. Ask for help with my work (if required) from an employer or member of production team

22. Get a colleague or peer to help me if I have difficulty interacting with others at my workplace

23. Get an employer or member of my production team to help me if I have difficulty interacting with others at my workplace

24. Network to secure future opportunities

2. Testing the reliability of the 'performing arts occupational self-efficacy scale'

Participants could select 'not applicable' to individual items on the self-efficacy scale which were not relevant to their careers. We wanted to examine whether there were any meaningful differences in mean self-efficacy scores between participants who completed all 24 items of the self-efficacy scale (N = 805), and those who completed fewer than 24 (N = 622). Mean self-efficacy scores for each participant were therefore calculated from the number of completed items only.

We did this by examining the differences between mean scores for the whole sample (N = 1427) and the subset of participants who completed all of the 24 items (N = 805). We also used multiple imputation (MI) to estimate the missing values for the participants who had completed fewer than 24 items (N= 622).

We found no meaningful differences between these different analyses, that is, by using the original raw data for all participants or analysis using multiple imputation for the missing values – which warrants confidence in our results.

There were no missing values in any of the other scales as unlike the self-efficacy scale, participants were required to complete every item on each scale, therefore no multiple imputation was necessary for other measures.

Cronbach's alpha of the occupational self-efficacy scale

Using original data (N=1427) Cronbach's alpha = .94
Using multiple imputation pooled estimate (N = 1427) Cronbach's alpha = .92
Using only participants who answered every item on the scale (N = 805) Cronbach's alpha = .92

Table 1. *This table shows mean self-efficacy scores calculated using the original data (including missing values), using the means of the pooled data from multiple imputation (MI), and using the means of only participants who completed all of the items on the self-efficacy scale.*

**Self-efficacy item**	**M (SD) of original sample (including missing values) N = 1427**	**M of sample with multiple imputation N = 1427**	**M (SD) of only ppts with no missing data N = 805**
1. Fully understand what I am required to do to be proactive in my career	7.5 (2.1)	7.5	7.3 (2.1)
2. Motivate myself to work (e.g. apply for roles, rehearse)	7.5 (2.1)	7.5	7.3 (2.1)
3. Fully understand all instructions given to me	8.3 (1.8)	8.3	8.1 (1.8)
4. Structure my time to manage my workload	7.3 (2.2)	7.3	7.1 (2.2)
5. Keep to external deadlines	8.6 (1.6)	8.6	8.4 (1.7)
6. Concentrate when at work	8.6 (1.6)	8.6	8.4 (1.7)
7. Remember information presented at work or in books	8.0 (1.8)	8.0	7.8 (1.8)
8. Take good notes during instruction from others	8.0 (1.9)	8.0	8.0 (1.9)
9. Independently study or research	8.1 (1.9)	8.1	8.0 (1.9)
10. Complete classes or workshops that I have signed up for	8.6 (1.8)	8.7	8.6 (1.8)
11. Participate in group exercises	8.0 (2.1)	8.0	8.1 (2.1)

12. Work with others to achieve a joint goal	8.8 (1.5)	8.8	8.7 (1.5)
13. Share my ideas in group discussions	8.2 (1.9)	8.2	8.1 (1.9)
14. Lead or coordinate my peers / colleagues in group work	7.5 (2.2)	7.5	7.4 (2.2)
15. Interview / audition for roles	7.5 (2.3)	7.5	7.4 (2.2)
16. Prepare for performances (this includes technical work, rehearsals, etc. as applicable)	8.8 (1.5)	8.8	8.7 (1.5)
17. Take part in performances	9.0 (1.5)	9.0	8.9 (1.5)
18. Make phone calls to people I don't know (for work-based purposes, e.g. to hire equipment)	6.8 (2.8)	6.8	6.6. (2.8)
19. Socialize with others in my workplace	7.5 (2.3)	7.5	7.4 (2.2)
20. Ask for help with my work (if required) from a colleague or peer	7.4 (2.3)	7.4	7.3 (2.2)
21. Ask for help with my work (if required) from an employer or member of production team	7.6 (2.2)	7.5	7.4 (2.2)
22. Get a colleague or peer to help me if I have difficulty interacting with others at my workplace	6.0 (2.8)	6.1	6.0 (2.7)
23. Get an employer or member of my production team to help me if I have difficulty interacting with others at my workplace	5.7 (2.9)	5.8	5.7 (2.8)
24. Network to secure future opportunities	5.6 (2.7)	5.6	5.7 (2.7)
**Total**	**7.8 (1.3)**	**7.7**	**7.6 (1.3)**

Table 2. *This table shows the comparison of self-efficacy (SE) columns between the correlation matrices calculated using the original mean SE scores and the correlation matrices calculated using the pooled scores from multiple imputation for SE. Eye-balling the figures in Tables 1 and 2 indicates very little difference between the two set of analyses, using the original scores or the MI scores does not affect the significance of any of the correlations.*

		Original SE (including missing values)	Pooled Multiple Imputation SE
SATQ	r_s	-.414**	-.413**
	Sig.	<.001	<.001
Self-efficacy	r_s	1.00	1.00
	Sig.		
WHOQOL physical domain	r_s	.333**	.330**
	Sig.	<.001	<.001
WHOQOL psychological domain	r_s	.458**	.451**
	Sig.	<.001	<.001
WHOQOL social domain	r_s	.329**	.323**
	Sig.	<.001	<.001
WHOQOL environment domain	r_s	.399**	.389**
	Sig.	<.001	<.001
PHQ-8	r_s	-.361**	-.354**
	Sig.	<.001	<.001
GAD-7	r_s	-.324**	-.314**
	Sig.	<.001	<.001
ASRS	r_s	-.319**	-.311**
	Sig.	<.001	<.001
Age	r_s	.237**	.222**
	Sig.	<.001	<.001
Gender	r_s	.01	.003
	Sig.	.73	.901

3. The influence of autistic participants on results

We wanted to examine whether the participants who reported a clinical diagnosis of autism had any significantly influential effects on our analyses, so we conducted correlational analyses and the extreme groups analysis (EGA) again without the 11 autistic participants (N = 1,416). Eye-balling the correlation coefficients in Table 3 below and Table 3 in the manuscript suggested no meaningful difference between our analyses using all participants (N = 1,427) and the analyses with autistic participants removed (N = 1,416).

Extreme Groups Analysis (EGA) excluding autistic participants

Professionals in the high autistic trait group were just as likely to have received support (25%) as those in the low autistic traits group (22%), χ^2 (1) = 2.74, p = .254. Members of the high autistic traits group were significantly more likely, however, to have needed support but not received it (39%) than members of the low autistic traits group (34%), χ^2 (1) = 7.51, p = .023. Analyses also revealed a significant group difference in terms of how many of them desired support in the future: professionals with high autistic traits were more likely to desire support in the future (48%) than those with low autistic traits (38%), χ^2 (1) = 11.50, p = .003.

Next, we examined the frequency of individuals in the high and low autistic traits groups scoring at clinically significant levels for depression, anxiety and ADHD traits. Professionals in the high autistic traits group were significantly more likely to meet clinically-significant thresholds on all of the measures (PHQ-8, GAD-7, ASRS) in comparison to the low autistic traits group (depression χ^2 (1) = 122.77, $p < .001$; anxiety χ^2 (1) = 66.54, $p < .001$; ADHD χ^2 (1) = 9.19, p = .002.

Table 3. *Correlation matrices for performing arts professionals without a diagnosis of autism, scores on occupational self-efficacy, SATQ, WHOQOL-BREF domains, PHQ-8, GAD-7, ASRS, age, and gender. Numbers with two asterisks ** beside them indicate a significant result.*

		SATQ	Self-efficacy	WHOQOL physical domain	WHOQOL psychological domain	WHOQOL social domain	WHOQOL environment domain	PHQ-8	GAD-7	ASRS	Age
SATQ	r_s	1.00									
	Sig.										
Self-efficacy	r_s	-.409**	1.00								
	Sig.	<.001									
WHOQOL physical domain	r_s	-.318**	.330**	1.00							
	Sig.	<.001	<.001								
WHOQOL psychological domain	r_s	-.399*	.456**	.587**	1.00						
	Sig.	<.001	<.001	<.001							
WHOQOL social domain	r_s	-.295**	.328**	.430**	.560**	1.00					
	Sig.	<.001	<.001	<.001	<.001						
WHOQOL environment domain	r_s	-.333**	.396**	.582**	.617**	.499**	1.00				
	Sig.	<.001	<.001	<.001	<.001	<.001					
PHQ-8	r_s	.380**	-.356**	-.583**	-.695**	-.433**	-.501**	1.00			

	Sig.	<.001	<.001	<.001	<.001	<.001	<.001				
GAD-7	r_s	.338**	-.321**	-.491**	-.639**	-.376**	-.486**	.782**	1.00		
	Sig.	<.001	<.001	<.001	<.001	<.001	<.001	<.001			
ASRS	r_s	.314**	-.314**	-.343**	-.413**	-.253**	-.382**	.480**	.478**	1.00	
	Sig.	<.001	<.001	<.001	<.001	<.001	<.001	<.001	<.001		
Age	r_s	-.134**	.236**	.031	.206**	.054*	.279**	-.277**	-.292**	-.289**	1.00
	Sig.	<.001	<.001	.241	<.001	.042	<.001	<.001	<.001	<.001	
Gender	r_s	.173**	.008	.031	.060	-.037	.048	-.083**	-.123**	-.062*	.165**
	Sig.	<.001	.754	.248	.024	.163	.069	.002	<.001	.020	<.001
